# Angiotensin II Alters Mitochondrial Membrane Potential and Lipid Metabolism in Rat Colonic Epithelial Cells

**DOI:** 10.3390/biom14080974

**Published:** 2024-08-09

**Authors:** Darby D. Toth, Christopher L. Souder, Sarah Patuel, Cole D. English, Isaac Konig, Emma Ivantsova, Wendi Malphurs, Jacqueline Watkins, Kaylie Anne Costa, John A. Bowden, Jasenka Zubcevic, Christopher J. Martyniuk

**Affiliations:** 1 Department of Physiological Sciences, Center for Environmental and Human Toxicology, College of Veterinary Medicine, University of Florida, Gainesville, FL 32611, USA; darby.toth@ufl.edu (D.D.T.); cksouders@gmail.com (C.L.S.II); sarahpatuel@ufl.edu (S.P.); coleenglish@ufl.edu (C.D.E.); isaac.konig@ufla.br (I.K.); eivantsova@ufl.edu (E.I.); wmalphur@ufl.edu (W.M.); jmecalwatkins@ufl.edu (J.W.); kcosta@ufl.edu (K.A.C.); john.bowden@ufl.edu (J.A.B.); 2 Department of Chemistry, Federal University of Lavras (UFLA), Lavras 37200-000, MG, Brazil; 3 Center for Hypertension and Precision Medicine, Department of Physiology and Pharmacology, The University of Toledo College of Medicine and Life Sciences, Block Health Science Bldg, 3000 Arlington Ave, Toledo, OH 43614, USA; jasenka.zubcevic@utoledo.edu; 4 University of Florida Genetics Institute, University of Florida, Gainesville, FL 32611, USA; 5 Interdisciplinary Program in Biomedical Sciences, Neuroscience, University of Florida, Gainesville, FL 32611, USA

**Keywords:** colonocytes, bioenergetics, lipidomics, lipids, mitochondria, angiotensin II

## Abstract

An over-active renin-angiotensin system (RAS) is characterized by elevated angiotensin II (Ang II). While Ang II can promote metabolic and mitochondrial dysfunction in tissues, little is known about its role in the gastrointestinal system (GI). Here, we treated rat primary colonic epithelial cells with Ang II (1–5000 nM) to better define their role in the GI. We hypothesized that Ang II would negatively affect mitochondrial bioenergetics as these organelles express Ang II receptors. Ang II increased cellular ATP production but reduced the mitochondrial membrane potential (MMP) of colonocytes. However, cells maintained mitochondrial oxidative phosphorylation and glycolysis with treatment, reflecting metabolic compensation with impaired MMP. To determine whether lipid dysregulation was evident, untargeted lipidomics were conducted. A total of 1949 lipids were detected in colonocytes spanning 55 distinct (sub)classes. Ang II (1 nM) altered the abundance of some sphingosines [So(d16:1)], ceramides [Cer-AP(t18:0/24:0)], and phosphatidylcholines [OxPC(16:0_20:5(2O)], while 100 nM Ang II altered some triglycerides and phosphatidylserines [PS(19:0_22:1). Ang II did not alter the relative expression of several enzymes in lipid metabolism; however, the expression of pyruvate dehydrogenase kinase 2 (*PDK2*) was increased, and *PDK2* can be protective against dyslipidemia. This study is the first to investigate the role of Ang II in colonic epithelial cell metabolism.

## 1. Introduction

The renin–angiotensin system (RAS) contributes to maintaining bodily homeostasis by regulating blood pressure, blood volume, electrolyte balance, and metabolism. However, the chronic activation of RAS can contribute to the pathogenesis of human conditions by promoting apoptosis, inflammation, and oxidative stress [1]. These cellular processes occur through both the Ang II type 1 (AT1R) and 2 receptors (AT2R) [2].

Ang II receptors are localized in mitochondria, and aberrant signaling can contribute to the pathophysiology of chronic diseases [3]. Though the role of Ang II in mitochondria is not completely clear, it has been demonstrated that activation of the RAS can impact oxidative respiration [4,5], potentially by decreasing mitochondrial membrane potential (MMP) [6] and decreasing the expression of components of the Krebs cycle and electron transport chain [7]. RAS plays a differing role in metabolism depending on the receptor subtype and site of action [8,9,10,11]. For example, the activation of Ang II receptor subtype 1 (AT1R) inhibited subcutaneous adipocyte lipolysis [12] and bicarbonate secretion in the GI tract [13], while activation of the Ang II receptor subtype 2 (AT2R) can oppose the AT1R action by enhancing the bicarbonate secretion by the duodenal mucosa [13]. This suggests a role for Ang II in GI function and lipid metabolism.

Mitochondria utilize lipid precursors to maintain homeostasis and cellular metabolism, and Ang II can promote metabolic and mitochondrial dysfunction in vascular smooth muscle cells and atrial myocytes in experimental animal models [14,15]. Dysfunctional mitochondria can generate reactive oxygen species (ROS), and Ang II-dependent release of mitochondrial ROS reportedly contributes to atherosclerosis lesion formation [16,17,18]. Moreover, Ang II can oxidize low-density lipoprotein (LDL), the accumulation of which contributes to dyslipidemia and hypertension [1]. RAS has been implicated in the modulation of GI function [19,20,21], and both GI and mitochondrial dysfunction are linked to metabolic disorders [22,23,24,25]. However, limited studies have examined the relationship between Ang II, mitochondrial bioenergetics, and lipid abundance in the GI tract. Here, we studied the effect of Ang II on rat epithelial colonocytes to gain a better understanding of its role in colonocyte metabolism, as colonocyte metabolism mediates homeostatic and dysbiosis transitions of gut microbiota [26]. This is important as gut dysbiosis is linked to chronic human illnesses and diseases (i.e., colorectal cancer, obesity, and diabetes).

## 2. Materials and Methods

### 2.1. Cell Culturing of Primary Colonocytes

Primary Rat Colon Intestinal Epithelial Cells (Cell Biologics Inc., Chicago, IL, USA, Cat# RN-6047) were grown aseptically in Cell Biologics Epithelial medium plus supplements (Cell Biologics, CAT# M6621) on T75 flasks coated with 3 mL of gelatin for 2 min (Cell Biologics, CAT# 6950). For passage, media were removed, and the flask was washed with 8 mL of HBSS and then incubated with 3 mL of TrypLE Express (Gibco, Thermo Fisher Scientific, Waltham, MA, USA, CAT# 12604039) at 37 °C for 2 min before the flask was checked under a microscope for detachment before 9 mL of media was added to quench the TrypLE. The media were then transferred to a 15 mL conical and centrifuged at 200× *g* for 5 min. The supernatant was then discarded, and the pellet was resuspended in full media downstream applications.

### 2.2. CellTiter-Glo^®^ Luminescent Cell Viability Assay

The CellTiter-Glo^®^ Luminescent Cell Viability Assay (Promega, Madison, WI, USA) was used to quantify metabolically active cells. Cells were seeded in a white 96-well plate at 10,000 cells/100 µL/well and allowed to attach overnight. The following day, cells were exposed at 1X concentration by adding 100 µL of 2X chemical to the corresponding well. The 72 h experiment was performed the same way but with a 100 µL media change at 24 and 48 h to renew Ang II exposure. Then, 24 or 72 h later, 100 µL was removed from each well using a multichannel. Next, 100 µL of cell titer glo reagent was added to each well. The plate was then mixed via an orbital shaker at 400 rpm for 30 s and incubated at room temperature for 10 min before luminescence was measured using a Biotek Synergy H1 plate reader (Agilent, Santa Clara, CA, USA). Epithelial cells were collected and treated with the corresponding concentration of Ang II in white 96-well culture plates for 24 and 48 h at 25 °C and 5% CO_2_. Experimental groups included “No cells” (to assess chemical interference with assay), media only, 0.1% DMSO solvent control, positive controls (TRIzol reagent for cell cytotoxicity and 10 mM of antimycin (AM) for shut down of ATP and oxidative phosphorylation), or one dose of Ang II at 10 nM, 50 nM, 100 nM, 500 nM, 1 μM, or 5 μM (n = 4/experimental group). Following treatment, CellTiter-Glo reagent was added to each well as per manufacturer’s instruction, and plates were incubated at room temperature for 10 min. This was followed by orbital shaking at 200 rpm for 2 min. The luminescence was recorded using a Synergy™ 4 Hybrid Microplate Reader (BioTek, Agilent, CA, USA).

### 2.3. Mitochondrial Membrane Potential

The MMP was measured using the mitochondrial Membrane Potential Kit (Sigma-Aldrich, St. Louis, MO, USA, MAK160-1KT). Briefly, epithelial cells were seeded in 90 μL at a density of 50,000 cells per well in black 96-well plates and treated with 10 μL of different 10X doses of Ang II (1, 10, 50, 100, 500, 1000, or 5000 nM) or either media, solvent control (0.1% DMSO), or positive control (4 and 8 μM of FCCP; see description below) (n = 4/treatment). The MMP assay proceeded as per our methods [27]. Fluorescence excitation/emission intensities of 540/590 nm (red) and 490/525 nm (green) were measured using a Synergy™ 4 Hybrid Microplate Reader (BioTek). Graphs represent the proportion of red/green fluorescence intensities per each well.

### 2.4. Mitochondrial Bioenergetics

Primary colon epithelial cells were seeded at 1.0 × 10^4^ cells per well in a Seahorse V7 cell culture plate overnight. Colon intestinal epithelial cells from WKY and SHR were seeded at 1.0 × 10^4^ cells per well in a Seahorse V7 cell culture plate overnight in cIEC growth media (n = 4 replicates/groups). Cells were treated for 72 h with either cIEC growth media only control or 1 µM of Ang II dissolved in cIEC growth media. The dose of Ang II was selected based on previous studies showing that mitochondrial function is affected in mouse Hl-1 atrial myocytes [14] and rat vascular smooth muscle cells [15]. The 72 h exposure time was selected because we aimed to induce ROS with elevated Ang II. Every 24 h, 80% of the media was renewed with freshly prepared chemicals, including Ang II. Following a 72 h treatment, live cells were washed with incubation media (97 mL of Seahorse XF DMEM, pH 7.4 [Agilent Technologies Inc., Santa Clara, CA, USA], 1 mL of 10 mM D-Glucose [Sigma-Aldrich], 1 mL of 1 mM pyruvate [Sigma-Aldrich], and 1 mL of 2 mM Ala-Gln [Sigma-Aldrich]), plated, and placed into the Seahorse Bioscience XFe24 Extracellular Flux Analyzer (Agilent, CA, USA). Background wells in the plate numbered n = 4. A bioenergetics profile was generated using specific uncoupling agents and mitochondrial toxicants. Each measurement cycle was as follows: 2:00 min mix, 1:00 min wait, and 3:00 min measure. There were 3 cycles for basal oxygen consumption rate. Each mitochondrial toxicant was then injected in sequential order (final concentration of 0.5 µM of oligomycin, 3 µM of carbonyl cyanide-4 (trifluoromethoxy)phenylhydrazone (FCCP), and 1 µM of antimycin A/100 nM Rotenone). Oligomycin is an inhibitor of ATP synthase and is used to assess oxygen consumption rate to estimate ATP production. FCCP is a mitochondrial uncoupler and is used to determine maximum respiration, and antimycin A is a complex III inhibitor used to inhibit oxygen-dependent ATP production. Cells were distributed equally across all wells. Oxygen Consumption Rate (OCR) was first corrected to total protein in the cells using a Pierce™ BCA Protein Assay Kit (Thermo Fisher Scientific, MA, USA). All statistical analyses were performed using ANOVA in GraphPad Prism 9.0 (Graphpad Software, Inc., La Jolla, CA, USA).

### 2.5. Lipidomics and Cell Exposure

Colon cells were seeded into T75 flasks and allowed to reach 50–60% confluence before the media were removed and replaced with 100 nM, 1 nM of Ang II, or media only (n = 4 or 5 replicate flasks per treatment (media control was n = 4, low Ang II was n = 5, and high Ang II was n = 4). Media changes were conducted every 24 h to renew the hormone. All procedures were conducted in a biological safety cabinet using aseptic techniques. After 72 h, to minimize the effect of time, 1 flask from each of the 3 experimental groups was passaged simultaneously. Cells were washed twice with 10 mL of DPBS, dissociated with 4 mL of TrypLE Express, and resuspended in 1 mL of ice-cold solution consisting of sterile filtered 0.15 g of ammonium acetate, 0.15 g of ammonium formate, 0.45 g of NaCl, and 1.1915 g of HEPES in 50 mL of DPBS. This cell suspension was centrifuged at 350× *g* for 5 min to obtain cell pellets. The supernatant was discarded, and cells were resuspended in 1 mL of ice-cold wash solution, then transferred to pre-weighed 1.7 mL tubes and centrifuged again at 300× *g* for 5 min before the wash solution was discarded. The cell pellet weight was determined, and the sample was frozen at −80 °C until LC-MS/MS analysis.

### 2.6. Lipids Analysis in Colonocytes

To quantify specific differences in individual lipid moieties, we employed an LC-MS/MS-based lipidomics approach that followed our previous work on cells [28]. For LC-MS/MS, cell pellets were resuspended in 200 µL of 1X Phosphate-Buffered Saline (PBS), vortexed for 1 min, and transferred to a glass vial (Fisherbrand, Fisher Scientific™, NH, USA, CAT # 14-959-35AA). In addition to cell pellets, 50 µL of SRM 1950 “Metabolites in Frozen Human Plasma” (National Institute of Standards and Technology, Gaithersburg, MD, USA) was used as a quality control sample, and additional extraction blanks (containing internal standards) and solvent blanks (no internal standards) were prepared in 500 µL of PBS. Next, 2 mL of chloroform, 1 mL of methanol, 300 µL of PBS (experimental groups only), and 10 µL of SPLASH^®^ LIPIDOMIX^®^ Mass Spec Internal Standard (Avanti, Birmingham, AL, USA, CAT # 330707) were added to each glass tube. Samples were then vortexed for 1 min and centrifuged at 1500× *g* and 4 °C for 5 min. The bottom organic layer, including the lipids, was collected in a new glass tube using a 9″ disposable glass Pasteur pipette (Fisherbrand, Fisher Scientific™, NH, USA, CAT # 13-678-20D) and latex bulb. Following this, a 2 mL chloroform wash was performed, repeating the extraction process to increase lipid recovery. The collected bottom layer of each sample was then evaporated until dryness using a stream of nitrogen gas. Dried lipid extracts were resuspended in 100 µL of 2-propanol and transferred to autosampler vials. Pools of each treatment group were generated by taking 25 µL of each replicate and combining them in a single autosampler vial.

Lipidomic analyses were conducted using a Thermo Vanquish UHPLC system paired with a Thermo Q-Exactive Orbitrap mass spectrometer. A Thermo Accucore C30 column was used (150 mm × 2.1 mm × 2.6 μm, Thermo Fisher Scientific, MA, USA, CAT# 03-452-139), and the mobile phase consisted of (A) 60:40 acetonitrile/water with 5 mM of ammonium formate and 0.1% formic acid and (B) 90:10 isopropanol/acetonitrile (*v*/*v*) with 5 mM of ammonium formate and 0.1% formic acid. The mobile phase gradient (40% B, linear increase to 55% B at 7 min, 65% B at 8 min, hold until 12 min, 95% B at 20 min, 100% B at 22 min, hold until 27 min, decrease to 40% B at 27.1 min, and hold at 100% B until 30 min), flow rate (0.25 mL/min), column compartment (45 °C), autosampler temperature (10 °C), and injection volume (10 µL) [28]. Full scans in both negative and positive ion modes were conducted for each sample with spray voltages of +/− 3.0 kV, depending on the mode. The scan range was *m*/*z* 100–1500 with a resolution of 70,000 at *m*/*z* 200. The source temperature was 300 °C. IE-Omics was used to create exclusion lists for iterative exclusions (IEs) as part of a data-dependent acquisition of tandem mass spectra run on each treatment group pool. In each IE, the top 10 sequentially abundant ions had stepped normalized collision energies of 20, 25, and 30; an isolation window of 1 Dalton; and a dynamic exclusion of 6 s. Integrated lipid identities were then generated using LipidMatch software (v3.5) [29]. Lipid peak areas were identified using the internal standards of a similar subclass or by retention time, and nanograms of lipid determined this way were then normalized to mg of sample for each sample to produce semi-quantitative values for each lipid. The quality control samples (SRM 1950) and extraction blank showed tight clustering in the principal component analysis (PCA) scores plot. The median percent coefficient of variation for all lipids in the SRM1950 group was 11.08% and 8.24% for triglycerides.

### 2.7. Gene Expression Analysis

Five separate flasks of cells were treated with 1, 10, 100, or 1000 nM of Ang II for 72 h as per the methods outlined above. Real-time PCR analysis followed our established methods [30]. Colon epithelial cells were briefly homogenized in TRIzol Reagent (Life Technologies, Carlsbad, CA, USA), and RNA was extracted as per the manufacturer’s protocol. A 2100 Bioanalyzer (Agilent) was used to evaluate RNA quality (RNA integrity values > 7). The cDNA synthesis proceeded with 750 ng RNA using the iScript (BioRad) kit followed by DNase treatment (Turbo DNase, Ambion, Thermo Fisher Scientific, MA, USA) as per the manufacturer’s instructions. No reverse transcriptase (NRT) controls (n = 4) and 2 no-template controls were prepared and included in all qPCR reactions. NRTs were constructed from a pool of 3 different RNA samples chosen at random, and this was carried out 3 times. The cDNA synthesis reaction proceeded as follows: 5 min at 25 °C, 30 min at 42 °C, and 5 min at 85 °C using a MyCycler™ Thermal Cycler (Bio-Rad, Hercules, CA, USA). The reaction mixture was then diluted at 1:20 with nuclease-free water and used for real-time PCR amplification.

Target genes were selected based on their role in apoptosis, mitochondrial oxidative respiration, oxidative stress, and lipid regulation. These genes included acetyl-CoA carboxylase (*acc*), acetyl-CoA carboxylase alpha (*acc1*), accetyl-CoA synthetase (*acs1*), adipose triglyceride lipase (*atgl*), acyl-coa dehydrogenase long chain (*acadl*), caspase 3 (*casp3*), catalase (*cat*), carnitine Palmitoyltransferase 1 (*cpt1*), carnitine palmitoyltransferase 2 (*cpt2*), diacylglycerol o-acyltransferase 1 (*dgat1*), diacylglycerol o-acyltransferase 2 (*dgat2*), fas cell surface death receptor (*fas*), hormone-sensitive lipase (*hsl*), peroxisome proliferator activated receptor gamma (*pparg*), pyruvate dehydrogenase kinase 1 (*pdk1*), pyruvate dehydrogenase kinase 2 (*pdk2*), pyruvate dehydrogenase kinase 4 (*pdk4*), stearoyl-coa desaturase 1 (*scd1*), superoxide dismutase 1 (*sod1*), superoxide dismutase 2 (*sod2*), and sterol regulatory element binding transcription factor 1 (*srebp-1c*). Supplemental Table S1 contains all the sequence information for target genes. The reference and target genes were measured using the CFX Connect Real-Time PCR Detection System (Bio-Rad, CA, U.S.A.) using SsoFast™ EvaGreen^®^ Supermix (BioRad, Hercules, CA, USA). Biological replicates (n = 4 per experimental group) were measured in duplicate. Two reference genes (ribosomal subunit 18 (*rps18*) and beta-actin (*β-actin*)) were used to normalize target expression. Normalized expression based upon the relative ∆∆Cq method was obtained for each target gene using CFX Manager™ software (v3.1) (baseline subtracted) [31]. Real-time PCR data followed recommendations outlined in the MIQE guidelines [32].

### 2.8. Statistical Analysis

All statistical analyses were conducted in GraphPad Prism (version 9). Data collected from the CellTiter-Glo^®^ Luminescent Cell Viability Assay, ApoTox-Glo™ Triplex Assay, MMP, and gene expression were analyzed using a one-way ANOVA followed by a Dunnett multiple comparison test to the solvent control (DMSO). Mitochondrial endpoints were calculated as per Seahorse XF Cell Mito Stress Test Kit User Guide (User Guide Kit 103015-100, Agilent) as per [33]. Significance for all assays was determined to be *p* < 0.05 (alpha = 0.05).

MetaboAnalyst 6.0 was used to statistically compare lipid concentrations in colonocytes. Lipid data were first normalized using quantile normalization on relative lipid abundance in each sample, followed by Log transformation (base 10). Auto-scaling (mean-centered/divided by the standard deviation of each variable) was then conducted to normalize data. A principal component analysis and a cluster analysis using the fast ward clustering algorithm were employed. A volcano plot was generated considering a fold change value of 2.0 (raw *p*-value < 0.05). An ANOVA was conducted to test for differences in lipid abundance between concentrations of AngII (FDR-corrected *p*-values).

## 3. Results

### 3.1. Cytotoxicity and Cell Viability

**Figure 1:**
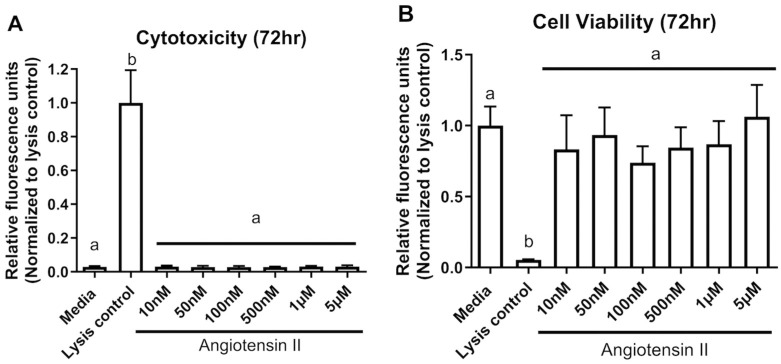
Cytotoxicity of Ang II to colonocytes at 72 h. (**A**) Cytotoxicity, (**B**) Cell viability. The lysis control was used as a positive control for the assay (induces cell death of colonocytes). The columns represent the mean relative fluorescence ± standard deviation. Different letters denote significant differences from the media-only control (One-way ANOVA, Dunnett multiple comparison test, n = 4/experiment, significance determined at *p* < 0.05).

**Figure 2:**
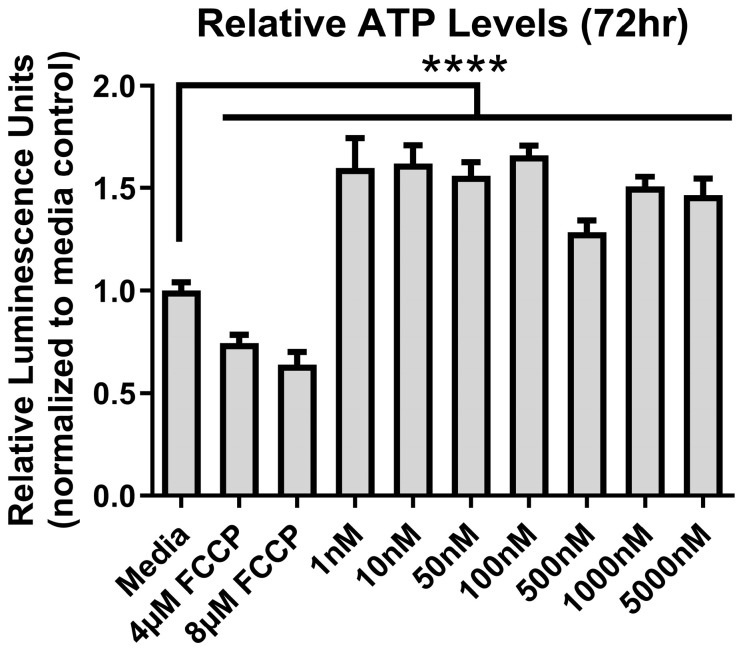
ATP levels after exposure to Ang II at 72 h. Carbonyl cyanide-4-phenylhydrazone (FCCP) was used as a positive control. Relative luminescence is graphed for each experimental group (horizontal bar represents mean relative luminescence ± standard deviation). Asterisks (****) denotes significant differences from the media-only control (One-way ANOVA followed by a Dunnett multiple comparison test, n = 4/experiment, significance determined at *p* < 0.001).

At 72 h, other than the controls, there was no change (*p* > 0.05) for both cytotoxicity (F _(7,24)_ = 100, *p* < 0.0001) and cell viability (F _(7,24)_ = 14, *p* < 0.0001) in colon epithelial cells exposed from 10 nM up to 5 µM of Ang II (Figure 1A,B). We also assessed metabolic output in the form of ATP using the CellTiter-Glo^®^ assay. ATP levels varied significantly at 72 h (F _(9,50)_ = 159.4, *p* < 0.0001) (Figure 2). There was a significant decrease in ATP levels following a 72 h treatment with the positive control FCCP, but there was a significant elevation in ATP at all concentrations of Ang II tested (*p* < 0.05) following a Dunnett’s post hoc test to the media control.

### 3.2. Mitochondrial Membrane Potential

**Figure 3:**
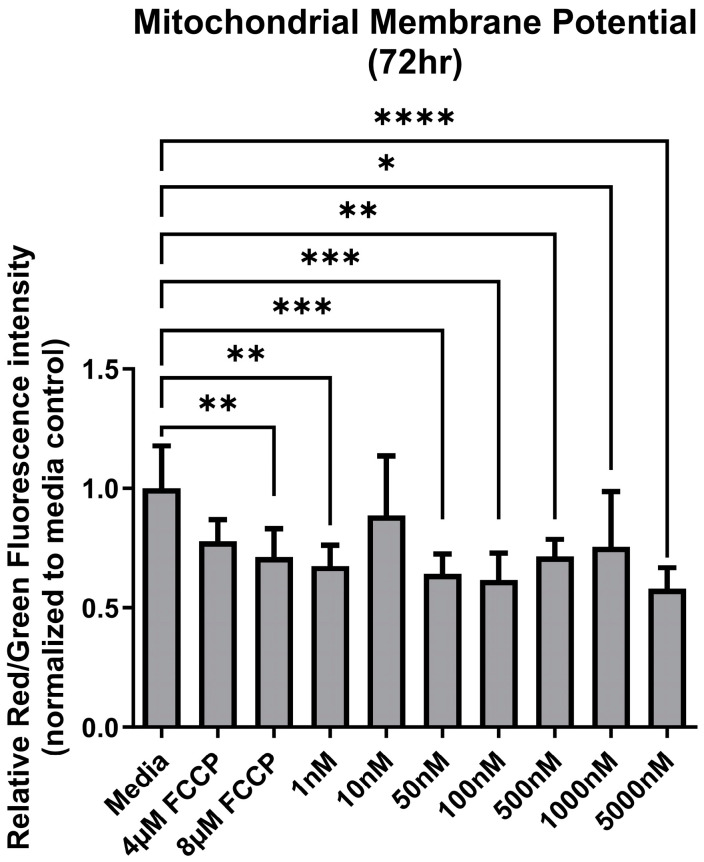
Mitochondrial membrane potential (MMP) after exposure to Ang II at 72 h. Carbonyl cyanide-4-phenylhydrazone (FCCP) was used as a positive control as it acts as an uncoupling agent for mitochondrial membranes. Relative fluorescence is based on the red/green signal intensity, and all data are normalized to the media-only control (mean relative fluorescence ± standard deviation). Asterisk denotes significant differences compared to the media-only control (one-way ANOVA followed by a Dunnett multiple comparison test, n = 4/experiment, significance determined at * *p* < 0.05, ** *p* < 0.01, *** *p* < 0.001, **** *p* < 0.0001).

Following a 72 h exposure to increasing concentrations of Ang II, the MMP of colon epithelial cells varied among treatment groups (F _(9,50)_ = 4.67, *p* = 0.0002) (Figure 3). There was a significant decrease in ATP levels following a 72 h treatment with the positive control FCCP. Similarly, at most concentrations of Ang II, MMP was significantly lower compared to the media (Figure 3).

### 3.3. Mitochondrial Bioenergetics

**Figure 4:**
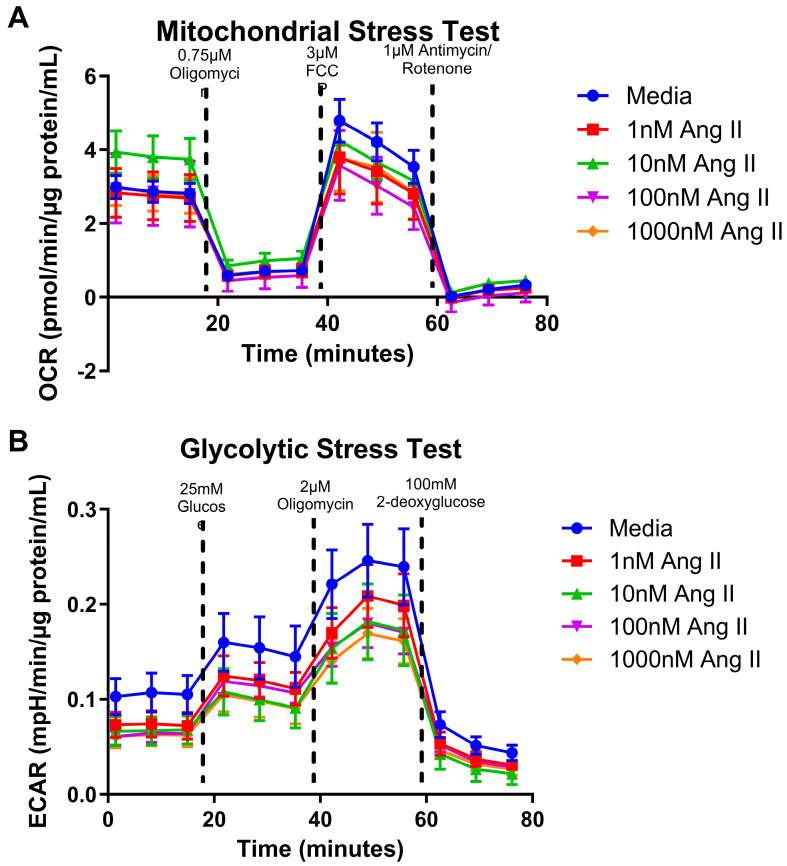
Normalized oxygen consumption rate and extracellular acidification rate for rat epithelial colonocytes after a 24 h exposure to Ang II. (**A**) Oxygen consumption rates over time (**B**) Acidification rates over time. Data are represented as mean ± standard deviation (one-way ANOVA followed by a Dunnett multiple comparison test, n = 4 replicates/groups, significance determined at *p* < 0.05).

Altered ATP levels and disruptions in MMP are indicators of metabolic dysfunction. Thus, we next investigated the metabolic capacity by measuring oxygen consumption rates (OCR) using a mitochondrial stress test and measuring the extracellular acidification rate (ECAR) using a glycolytic stress test in colonocytes treated with Ang II. We found that increasing concentrations of Ang II did not affect the mitochondrial (Figure 4A) or glycolytic stress test in treated colonocytes (Figure 4B) (*p* > 0.05). We concluded that treatment with Ang II for 72 h did not influence the mitochondrial bioenergetic profiles of colonocytes.

### 3.4. Lipid Abundance in Ang II Cultured Colonocytes

**Figure 5:**
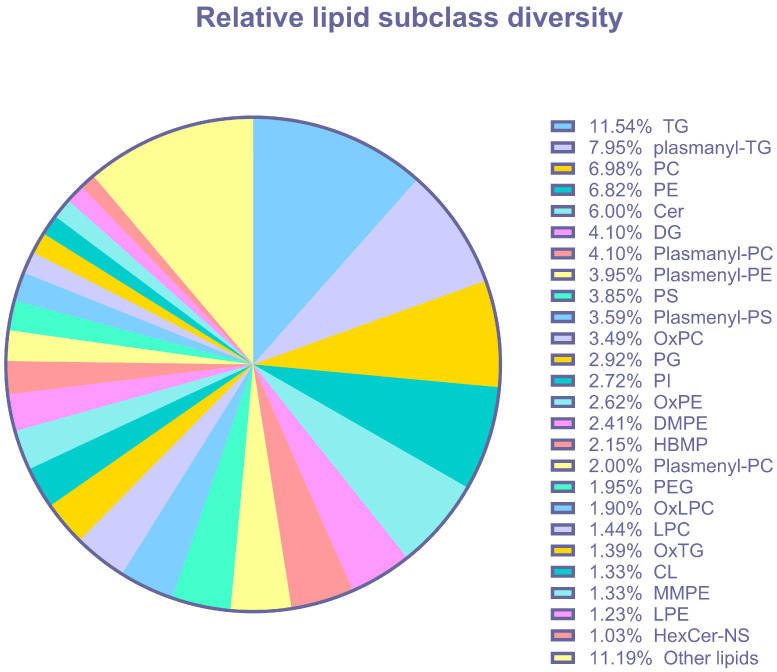
Lipid abundance and categorical classification of lipids in rat epithelial colonocytes (all lipids detected in all treatments). Abbreviations: triglycerides (TG), plasmanyl-TG (plasmanyl-triglycerides), phosphatidylcholine (PC), phosphatidylethanolamines (PE), ceramide (Cer), diacylglycerol (DG), plasmanyl-PC (plasmanyl-phosphatidylcholine), plasmenyl-PE (plasmenyl- phosphatidylethanolamines), phosphatidylserines (PS), plasmenyl-PS (plasmenyl-phosphatidylethanolamines), oxidized phosphatidylcholines (OxPC), phosphatidylglycerol (PG), phosphoinositide (PI), oxidized phosphatidylethanolamines (PE), dimethyl-phosphatidylethanolamine (DMPE), hemibismonoacylglycerophosphate (HBMP), plasmenyl-PC (plasmenyl-phosphatidylcholine), polyethylene glycol (PEG), oxidized lysophosphatidylcholines (OxLPC), lysophosphatidylcholines (LPC), oxidized triglycerides (OxTG), cardiolipins (CL), monomethyl-phosphatidylethanolamine (MMPE), lysophosphatidylethanolamine (LPE), and glucosylceramide non-hydroxyfatty acid-sphingosine (HexCer-NS).

**Figure 6:**
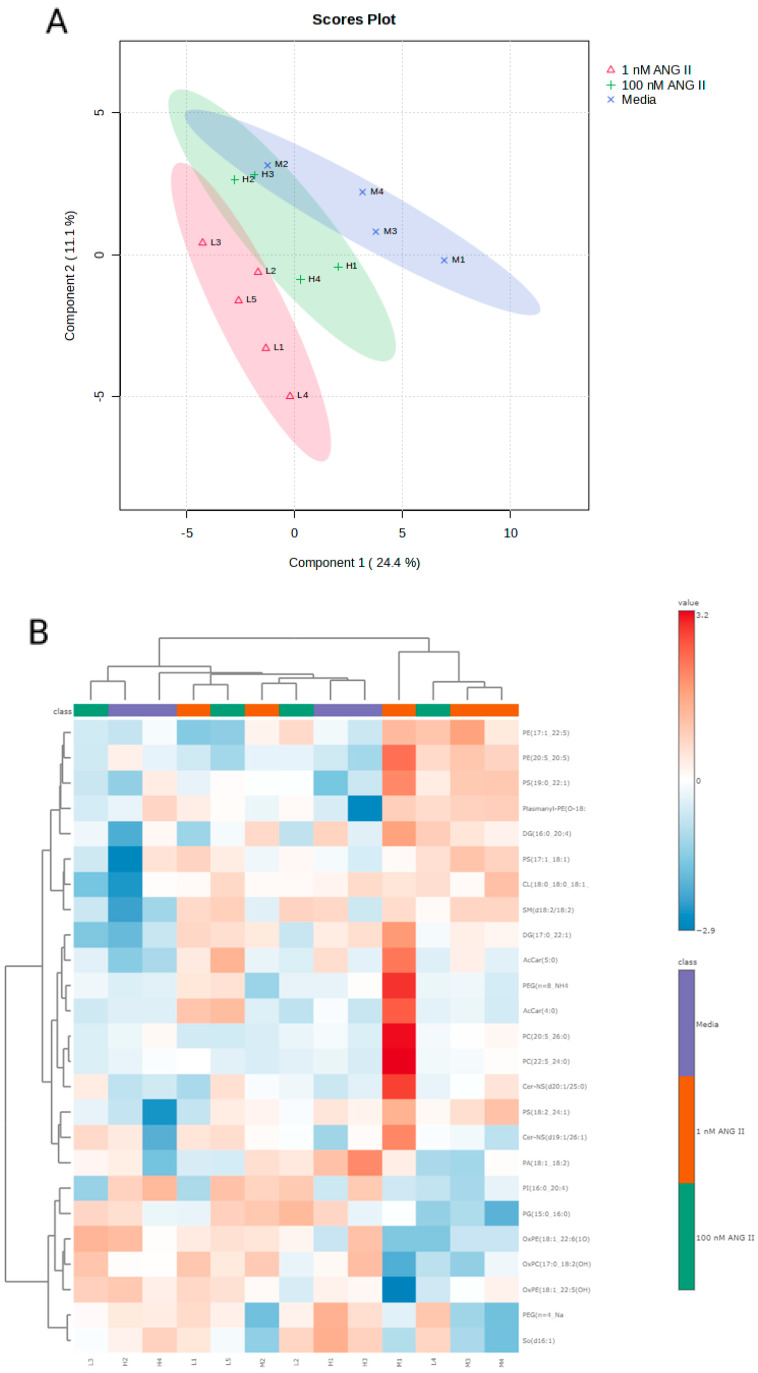
(**A**-Top graph) Principal component analysis scores plot for rat colonocyte lipids with each point representing the lipids in a single sample, the ellipses representing the 95% confidence interval, and the colored groups representing the three different treatments (blue = control, red = low Ang II, and green = high Ang II). (**B**-bottom graph) Heatmap showing significant changes in the levels of lipids following exposure to Ang II. Data were subjected to ANOVA followed by Fisher’s least significant difference method (Fisher’s LSD), and significant changes were set at *p* < 0.05.

As mitochondria are responsible for lipid metabolism, we performed untargeted lipidomics to elucidate the effects of Ang II on colonic lipid metabolism. All data, including abundances corrected to sample weight, as well as the categorical classification of lipids in colonocytes, are provided in Supplementary Data S1. Untargeted lipidomics detected 1949 lipids, categorized into 55 distinct (sub)classes of lipids that included acylcarnitines (AcCar), cardiolipins (CL), ceramides (CE), phosphatidylinositols (PI), phosphatidylserines (PS), sphingomyelins (SM), cholesterol (CH), and triglycerides (TG) (Figure 5, Supplementary Data S1). The most abundant lipid classes in colonocytes were TGs and plasmanyl-TGs, followed by phosphatidylcholines (PCs). A 3D PCA plot revealed a separation between experimental groups (Figure 6A). A cluster analysis indicated that the lipid response to 1 nM of Ang II was most different from the media-only control and 100 nM of Ang II (Figure 6B). The heatmap depicts the lipids that have been altered in abundance by Ang II. Data were first subjected to an ANOVA followed by Fisher’s least significant difference test (Fisher’s LSD). These top lipids were then clustered by their relative abundance to better reveal how Ang II affects the lipidome of colonocytes.

**Figure 7:**
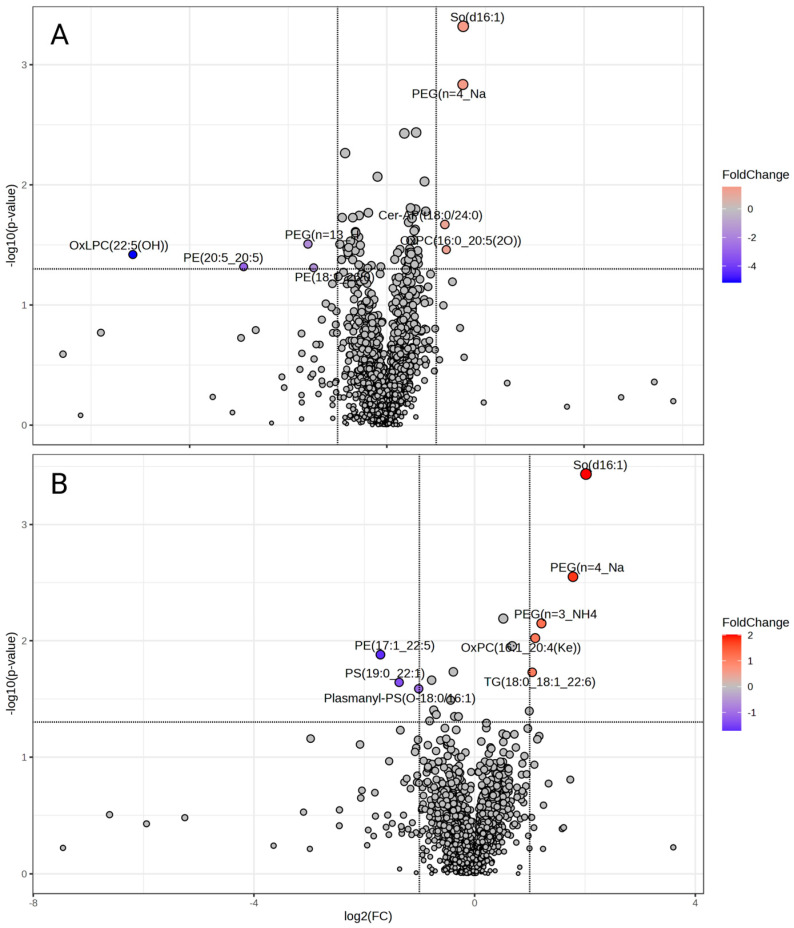
Volcano plots of (**A**) 1 nM of Ang II and (**B**) 100 nM of Ang II and the differentially abundant lipids (*p* < 0.05) outlined in red and blue.

**Figure 8:**
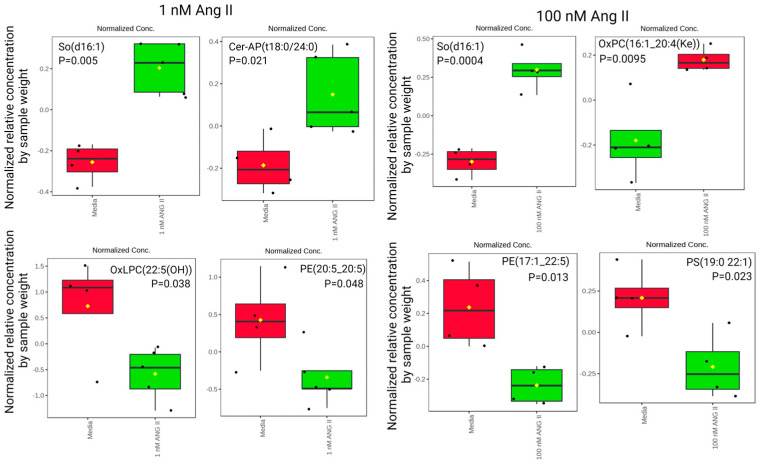
Relative concentrations of lipid abundance by sample weight in rat epithelial colonocytes exposed to 1 nM of Ang II (**left panel**). The most abundant lipids measured include So(d16:1) and Cer-AP(t18:0/24:0). Abbreviations: sphingosine (So), alpha-hydroxy-fatty acid phytosphingosine ceramide (Cer-AP), oxidized lysophosphatidylcholines (OxLPC), and phosphatidylethanolamines (PE). Relative concentrations of lipid abundance by sample weight in rat epithelial colonocytes exposed to 100 nM of Ang II (**right panel**). The most abundant lipid measured was So(d16:1). Abbreviations: sphingosine (So), oxidized phosphatidylcholines (OxPC), phosphatidylethanolamines (PE), and phosphatidylserines (PS). The black dots represent the metabolite levels in all samples, and the yellow diamond represents the average value.

A volcano plot was generated to visualize significant differences in lipid abundances (ANOVA, *p* < 0.05). Figure 7A illustrates significant differences in lipids following treatment with 1 nM of Ang II, and Figure 7B highlights significant differences following treatment with 100 nM of Ang II compared to control media. We observed elevated So(d16:1) and Cer-AP(t18:0/24:0) and downregulated OxLPC(22:5(OH)) and PE(20:5_20:5) levels following 1 nM of Ang II treatment (Figure 8, left panel). Moreover, the 100 nM treatment elevated So(d16:1) and OxPC(16:1_20:4(Ke)) and decreased PE(17:1_22:5) and PS(19:0_22:1) (Figure 8, right panel). The complete list of lipids that were different in abundance after Ang II treatment is presented in Supplementary Data S1.

### 3.5. Expression Levels of Lipid and Metabolic-Related Genes in Colonocytes

**Figure 9:**
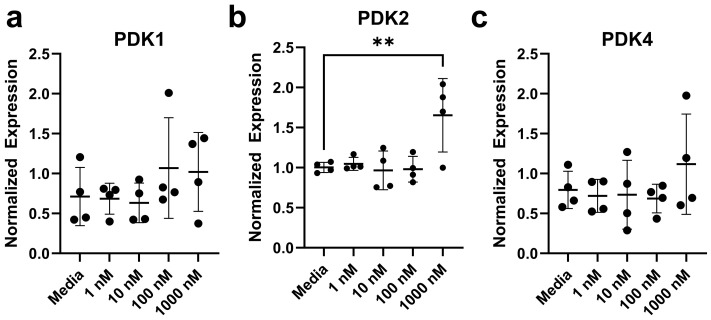
Relative gene expression for rat colonocytes after exposure to Ang II. (**a**) *PDK1,* (**b**) *PDK2*, (**c**) *PDK4*. Data are represented as mean ± standard deviation. Asterisks (**) denote significant differences from the media-only control (data were evaluated using a Mann–Whitney U test, n = 4/experiment, significance determined at *p* < 0.01).

Based on the previous research, we confirm our suspicion that Ang II can impact metabolic-related genes in colonocytes. There were 21 transcripts measured in colonocytes (Supplemental Table S1). We did not observe any difference in the expression of 20 out of 21 genes tested, each presented in Figure 9 and Supplemental Figures S1–S3. The relative expression level of pyruvate dehydrogenase kinase isoform 2, *PDK2* (F _(4,15)_ = 4.70, *p* = 0.0117), was increased in rat colonocytes treated with 1000 nM of Ang II, while the other isoforms measured (*PDK1* and *PDK4*) were unchanged (*p* < 0.05) (Figure 9).

## 4. Discussion

While a plethora of studies link Ang II to mitochondrial dysfunction and oxidative stress in the heart and kidney in conditions of hypertension and diabetes [18,34,35], there has been little focus to date on the gut epithelium. Ang II receptors have been found in the mucosal and submucosal layers of rat small intestine [36]; in all layers of Mongolian gerbil gastric wall [37]; and in several regions of the human colon [38], esophagus [39], gastric wall [40], and small intestine [41]. Thus, the direct effects of Ang II on the GI tract, specifically on epithelial cell function and its implications for conditions associated with elevated RAS, warrant study. As such, the objectives of this study were to determine the effect of Ang II on colonocyte metabolism to better understand the relationship between Ang II and the GI system.

Our study, to the best of our knowledge, is the first to suggest a role for Ang II in mitochondrial function and specifically MMP in rat colonocytes. Damaged mitochondria are characterized by inhibited electron transport that leads to the generation of ROS, which can affect the membrane composition and fluidity, thus hindering the mitochondrial capacity to generate optimum MMP [42]. Consequently, inhibition of Ang II signaling can improve mitochondrial function [43]. Our study reports a deleterious effect of Ang II on MMP but no effect on cell viability, suggesting no mitochondrial DNA damage that may lead to subsequent apoptosis [44,45]. This suggests a physiologic function for Ang II in rat colonocytes, at least at concentrations used in our current study.

Others have reported Ang II’s effects on oxygen consumption in mouse HL-1 atrial myocytes [14] and rat vascular smooth muscle cells [15]. While we did not observe a change in oxidative phosphorylation or glycolytic capacity, Ang II treatment did elevate ATP, suggesting metabolic action independent of the mitochondria. This switch could potentially be mediated by inhibition of the pyruvate dehydrogenase complex (PDC), which converts pyruvate to acetyl-CoA [46]. Inactivation of PDC activity is facilitated by several isoforms of pyruvate dehydrogenase kinase (PDK) [47] and may lead to the activation of alternative pathways to metabolize pyruvate in which the conversion of glucose to lactate, as well as ATP generation via glycolysis, are enhanced [48]. While we did not observe significant changes in glycolytic capacity, higher concentrations of Ang II tended to decrease glycolysis in our colonocytes. This, and the observed elevation in *PDK2* transcription following Ang II treatment, may be responsible for the ATP generation we see in our model, as PDK2 can inhibit PDC and is key in mediating rapid changes in glucose, pyruvate, and lactate levels in many tissues [48].

PDK2 may also contribute to lipid metabolism. Others have reported PDK2 activation leading to decreased PDC activity and ketogenesis and increased pyruvate levels and the levels of Krebs cycle intermediates in mice fed high-fat diets [46,49]. In contrast, these effects are ameliorated in mice that are deficient in PDK2 globally or in adipose-specific tissues [46], suggesting a role for PDK2 in hyperlipidemia. The RAS also plays an important role in lipid metabolism as the inhibition of RAS has reportedly led to the improvement in kidney lipid network architecture in diabetic mice [50], and alterations in both the lipid metabolism and mitochondrial function contribute to the development of cardiovascular disease and metabolic syndrome [10,18,51]. Here, we report the first evidence linking PDK2 with RAS, mitochondrial dysfunction, and dyslipidemia in rat colonocytes, and further studies are warranted to investigate direct causation.

We observed the elevation of sphingosine (d16:1) (i.e., So(d16:1)) following exposure to increasing concentrations of Ang II. So(d16:1) is present in human plasma in its free form following dietary saturated fatty acid intake [52] and has been linked with various aspects of cardiovascular diseases [53]. In addition, we found decreased phosphatidylethanolamine (PE) and alterations in phosphatidylserine (PS) with Ang II treatment. Both lipids compose cell membranes and are involved in biological processes, including apoptosis and cell signaling [54]. PE is the second most abundant phospholipid in mammalian membranes and is enriched in mitochondrial inner membranes [55]. Its content affects mitochondrial function and oxidative phosphorylation [56], which are both linked to cardiovascular disease. We observed no changes in oxidative phosphorylation, but we did see a perturbance in MMP, suggesting that Ang II may affect mitochondrial membrane function in rat colonocytes. We also measured the expression levels of several lipid enzymes following treatment with Ang II; however, these transcripts were unaltered by Ang II, suggesting that transcriptional modulation may not be an important mechanism for Ang II-mediated effects on the lipidome. Further studies are needed to translate our findings to the more general effects on cardiovascular health.

In summary, the role of RAS in metabolic function has been a major focus in recent decades in cardiovascular disease [1,51,57], but the role of the GI tract remains unclear. In the current study, for the first time, we demonstrate the deleterious effects of increasing concentrations of Ang II on the colonic primary epithelial cell mitochondrial function and lipid abundances. However, one limitation is that we are unsure if these effects are mediated via AT1R or AT2R receptors; thus, it is still uncertain how these in the GI tract may contribute to conditions associated with elevated RAS.

## Data Availability

The original contributions presented in the study are included in the article/Supplementary Material, further inquiries can be directed to the corresponding author.

## Supplementary Materials

The following supporting information can be downloaded at: https://www.mdpi.com/article/10.3390/biom14080974/s1, Figure S1: Relative gene expression of lipid-related transcripts in rat epithelial colonocytes after exposure to angiotensin II; Figure S2: Relative gene expression of apoptosis-related transcripts in rat epithelial colonocytes after exposure to angiotensin II; Figure S3: Relative gene expression of oxidative stress-related transcripts in rat epithelial colonocytes after exposure to angiotensin II; Table S1: Primers used for real-time PCR analysis [58,59,60,61,62,63,64,65,66]. Data S1: Lipidomics dataset, normalized.
