# Supplementary material for: Angiotensin II Alters Mitochondrial Membrane Potential and Lipid Metabolism in Rat Colonic Epithelial Cells
Source: Biomolecules. 2024 Aug 9;14(8):974. doi: 10.3390/biom14080974 (PMC11353208; doi:10.3390/biom14080974)
Supplement: Supplementary file 1 [file biomolecules-14-00974-s001.zip › Supplemental Figures.docx]

**Angiotensin II alters Mitochondrial Membrane Potential and Lipid Metabolism in Rat Colonic Epithelial Cells**

**Darby D. Toth ^1,#^, Christopher L. Souder II ^1,#^, Sarah Patuel ^1^, Cole D. English ^1^, Isaac Konig ^1,4^, Emma Ivantsova ^1^, Wendy Malphurs ^1^, Jackie Watkins ^1^, Kaylie Anne-Costa ^1^, John A. Bowden ^1^, Jasenka Zubcevic ^5^, Christopher J. Martyniuk ^1,2,3,*^**

^1^ Department of Physiological Sciences and Center for Environmental and Human Toxicology, College of Veterinary Medicine, University of Florida, Gainesville, Florida 32611, USA

^2^ University of Florida Genetics Institute, University of Florida, Gainesville, FL 32611, USA

^3^ Interdisciplinary Program in Biomedical Sciences, Neuroscience, University of Florida, Gainesville, FL 32611, USA

^4^ Department of Chemistry, Federal University of Lavras (UFLA), Minas Gerais, Brazil

^5^ Center for Hypertension and Precision Medicine, Department of Physiology and Pharmacology, The University of Toledo College of Medicine and Life Sciences, Block Health Science Bldg, 3000 Arlington Ave, Toledo, OH, 43614, USA

***** Correspondence: [cmartyn@ufl.edu](mailto:cmartyn@ufl.edu) (2187 Mowry Rd. Bldg 471, PO Box 110885, University of Florida)

**#** Authors contributed equally




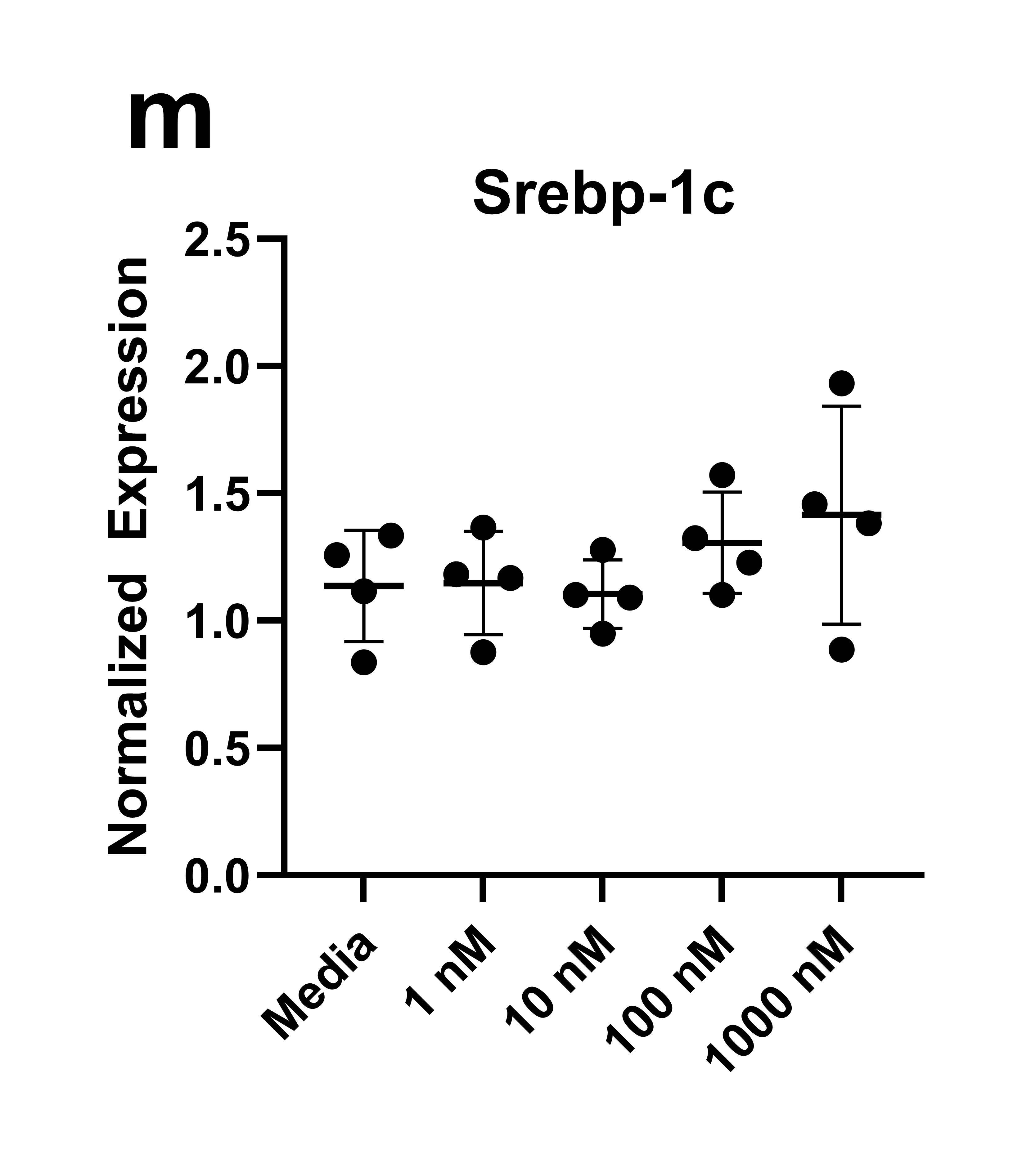


**Supplemental Figure S1.** Relative gene expression of lipid-related transcripts in rat epithelial colonocytes after exposure to angiotensin II. (A) *cpt1,* (B) *cpt2,* (C) *dgat1,* (D) *dgat2,* (E) *atg1,* (F) *acadl,* (G) *acc,* (H) *scd1*, (I) *pparg,* (J) *hsl*, (K) *acs1*, (L) *acc1*, and (M) *srepb-1c*. Data are represented as mean ± standard deviation. Asterisk denotes significant differences from the media-only control (data were evaluated using a Mann-Whitney U test, n=4/experiment, significance determined at p<0.05).


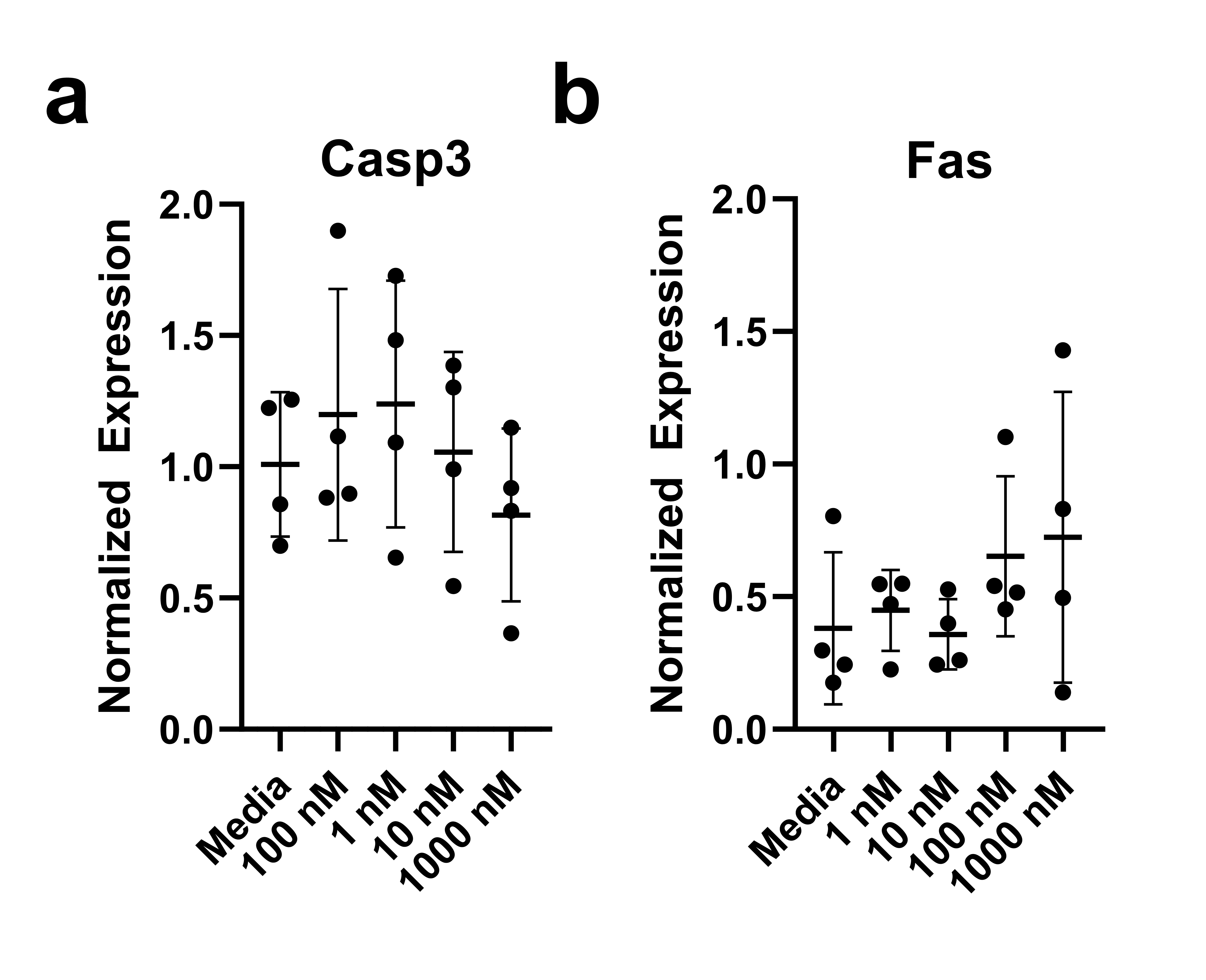


**Supplemental Figure S2.** Relative gene expression of apoptosis-related transcripts in rat epithelial colonocytes after exposure to angiotensin II. (A) *casp3* and (B) *fas.* Data are represented as mean ± standard deviation. Asterisk denotes significant differences from the media-only control (data were evaluated using a Mann-Whitney U test, n=4/experiment, significance determined at p<0.05).


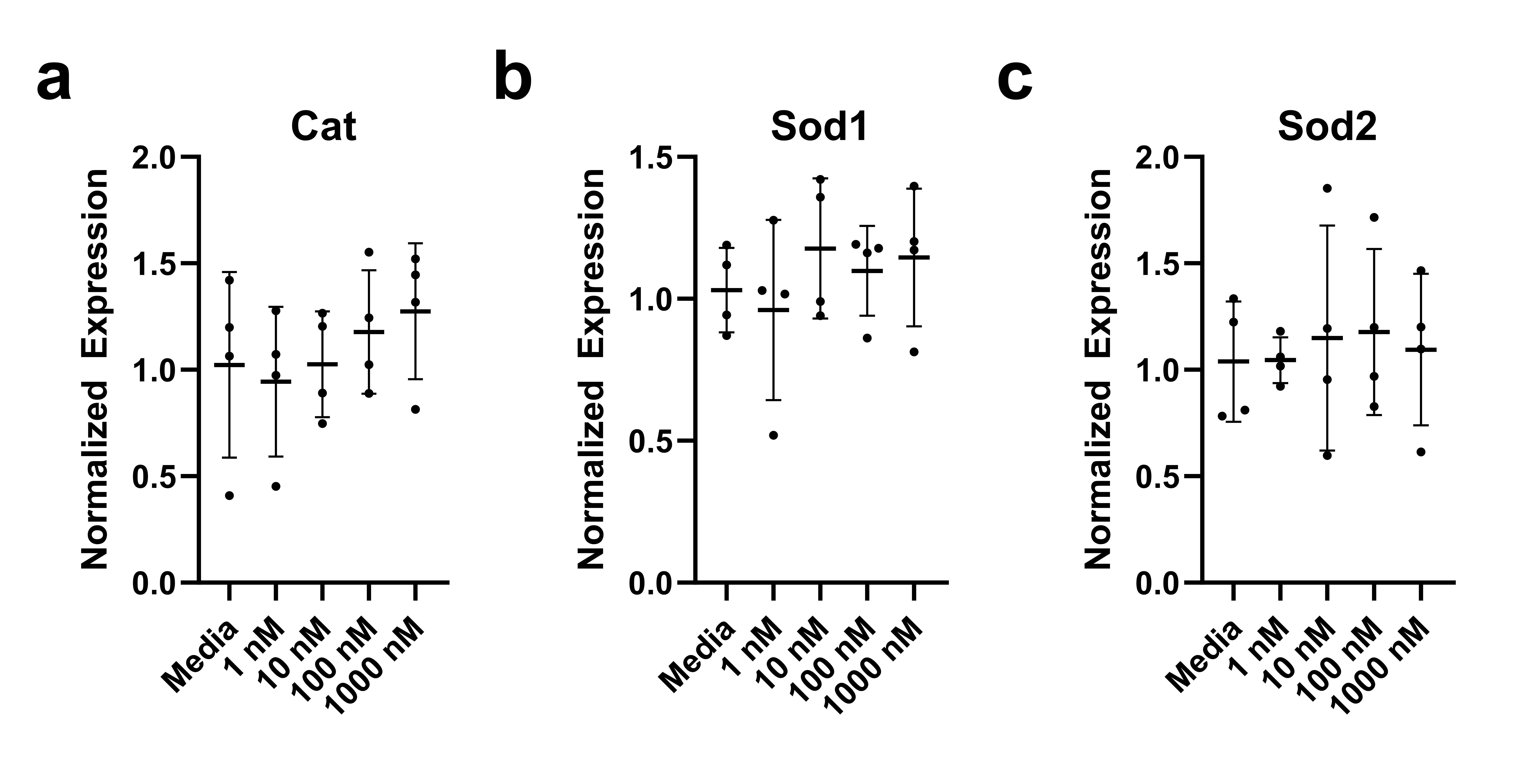


**Supplemental Figure S3.** Relative gene expression of oxidative stress-related transcripts in rat epithelial colonocytes after exposure to angiotensin II. (A) *cat,* (B) *sod1,* and (C) *sod2*. Data are represented as mean ± standard deviation. Asterisk denotes significant differences from the media-only control (data were evaluated using a Mann-Whitney U test, n=4/experiment, significance determined at p<0.05).
