# Supplementary material for: Angiotensin II Alters Mitochondrial Membrane Potential and Lipid Metabolism in Rat Colonic Epithelial Cells
Source: Biomolecules. 2024 Aug 9;14(8):974. doi: 10.3390/biom14080974 (PMC11353208; doi:10.3390/biom14080974)
Supplement: Supplementary file 1 [file biomolecules-14-00974-s001.zip › Supplemental Table.docx]

**Angiotensin II alters Mitochondrial Membrane Potential and Lipid Metabolism in Rat Colonic Epithelial Cells**

**Darby D. Toth ^1,#^, Christopher L. Souder II ^1,#^, Sarah Patuel ^1^, Cole D. English ^1^, Isaac Konig ^1,4^, Emma Ivantsova ^1^, Wendy Malphurs ^1^, Jackie Watkins ^1^, Kaylie Anne-Costa ^1^, John A. Bowden ^1^, Jasenka Zubcevic ^5^, Christopher J. Martyniuk ^1,2,3,*^**

^1^ Department of Physiological Sciences and Center for Environmental and Human Toxicology, College of Veterinary Medicine, University of Florida, Gainesville, Florida 32611, USA

^2^ University of Florida Genetics Institute, University of Florida, Gainesville, FL 32611, USA

^3^ Interdisciplinary Program in Biomedical Sciences, Neuroscience, University of Florida, Gainesville, FL 32611, USA

^4^ Department of Chemistry, Federal University of Lavras (UFLA), Minas Gerais, Brazil

^5^ Center for Hypertension and Precision Medicine, Department of Physiology and Pharmacology, The University of Toledo College of Medicine and Life Sciences, Block Health Science Bldg, 3000 Arlington Ave, Toledo, OH, 43614, USA

***** Correspondence: [cmartyn@ufl.edu](mailto:cmartyn@ufl.edu) (2187 Mowry Rd. Bldg 471, PO Box 110885, University of Florida)

**#** Authors contributed equally

**Supplemental Table S1.** Primers used for real-time PCR analysis.

| **Gene name** | **Gene Symbol** | **Forward (5' to 3')** | **Reverse (5' to 3')** | **Reference** |
| --- | --- | --- | --- | --- |
| acyl-CoA dehydrogenase long chain | *acadl* | TTTCCTCAGAGCATGACATTTT | GCCAGCTTTTTCCCAGAGCT | Jernberg et al. 2017. |
| acetyl-CoA carboxylase | *acc* | GGACCACTGCATGGAATGTTAA | TGAGTGACTGCCGAAACATCTC | Alberdi et al., 2011 |
| acetyl-CoA carboxylase 1 | *acc1* | ATTGTGGCTCAAACTGCAGGT | GCCAATCCACTCGAAGACCA | Choi et al., 2007 |
| acetyl-CoA synthetase | *acs1* | TGACAGCACAAGCACAAAAC | TTCCACTCGTGAAGCAAATC | Jernberg et al. 2017 |
| adipose triglyceride lipase | *atgl* | CACTTTAGCTCCAAGGATGA | TGGTTCAGTAGGCCATTCCT | Alberdi et al., 2011 |
| beta-actin | *bactin* | ACGAGGCCCAGAGCAAGAG | GGTGTGGTGCCAGATCTTCTC | Alberdi et al., 2011 |
| caspase 3 | *casp3* | GTGGAACTGACGATGATATGGC | CGCAAAGTGACTGGATGAACC | He et al., 2018 |
| catalase | *cat* | ATGGCTTTTGACCCAAGCAA | CGGCCCTGAAGCTTTTTGT | Chan et al., 2006 |
| carnitine palmitoyl transferase 1 | *cpt1* | TTGCACGAGGGAAAAATAAGC | CCAACGTCACGAAGAACGC | Choi et al., 2007 |
| carnitine palmitoyl transferase 2 | *cpt2* | GAGCCCCTAGTAGGCCCTTA | AGGCTTCTGTGCATTGAGGT | Carnevali et al., 2012 |
| diacylglycerol O-acyltransferase 1 | *dgat1* | CAGACCAGCGTGGGCG | GAACAAAGAGTCTTGCAGACGATG | Choi et al., 2007 |
| diacylglycerol O-acyltransferase 2 | *dgat2* | GGAACCGCAAAGGCTTTGTA | AATAGGTGGGAACCAGATCAGC | Choi et al., 2007 |
| fas cell surface death receptor | *fas* | AGCCCCTCAAGTGCACAGTG | TGCCAATGTGTTTTCCCTGA | Alberdi et al., 2011 |
| hormone-sensitive lipase | *hsl* | AATGACACAGTCGCTGGTGGCG | TGCCACACCCAAGAGCTGACCT | Wojcik et al., 2007 |
| pyruvate dehydrogenase kinase 1 | *pdk1* | ACCATGCAGACAAAGGCGTTT | TATACACAGGGAGTCTTTCGATG | Nakai et al., 2000 |
| pyruvate dehydrogenase kinase 2 | *pdk2* | GCCACGAGTCCAGCCTCACTC | GACAGGCAGGCGCTCCACT | Nakai et al., 2000 |
| pyruvate dehydrogenase kinase 4 | *pdk4* | ATCAAGAAAACCGCCCTTTCCT | GGGGAGTTTTTCTATAGACTCAGAT | Nakai et al., 2000 |
| peroxisome proliferator activated receptor gamma | *pparg* | ATTCTGGCCCACCAACTTCGG | TGGAAGCCTGATGCTTTATCCCCA | Alberdi et al., 2011 |
| stearoyl-CoA desaturase 1 | *scd1* | AATCACTGTAGATCTGATGACCTGGA | GCATCTGTGACCTGGAAAAATAAA | Choi et al., 2007 |
| superoxide dismutase 1 | *sod1 (Cu/Zn SOD)* | CACTCTAAGAAACATGGCG | CTGAGAGTGAGATCACACG | Chan et al., 2006 |
| superoxide dismutase 2 | *sod2 (Mn SOD)* | TTCAGCCTGCACTGAAG | GTCACGCTTGATAGCCTC | Chan et al., 2006 |
| sterol regulatory element binding transcription factor 1 | *srebp-1c* | GCGGACGCAGTCTGGG | ATGAGCTGGAGCATGTCTTCAAA | Alberdi et al., 2011 |
| ribosomal 18s | *rps18* | AAGTTTCAGCACATCCTGCGAGTA | TTGGTGAGGTCAATGTCTGCTTTC | Long et al., 2019 |

**References:**

Alberdi, G., Rodríguez, V. M., Miranda, J., Macarulla, M. T., Arias, N., Andrés-Lacueva, C., & Portillo, M. P. (2011). Changes in white adipose tissue metabolism induced by resveratrol in rats. Nutrition & metabolism, 8(1), 29. https://doi.org/10.1186/1743-7075-8-29

Carnevali, L. C., Jr, Eder, R., Lira, F. S., Lima, W. P., Gonçalves, D. C., Zanchi, N. E., Nicastro, H., Lavoie, J. M., & Seelaender, M. C. (2012). Effects of high-intensity intermittent training on carnitine palmitoyl transferase activity in the gastrocnemius muscle of rats. *Brazilian journal of medical and biological research = Revista brasileira de pesquisas medicas e biologicas*, *45*(8), 777–783. https://doi.org/10.1590/s0100-879x2012007500105

Chan, S. H., Tai, M. H., Li, C. Y., & Chan, J. Y. (2006). Reduction in molecular synthesis or enzyme activity of superoxide dismutases and catalase contributes to oxidative stress and neurogenic hypertension in spontaneously hypertensive rats. Free radical biology & medicine, 40(11), 2028–2039. https://doi.org/10.1016/j.freeradbiomed.2006.01.032

Choi, C. S., Savage, D. B., Kulkarni, A., Yu, X. X., Liu, Z. X., Morino, K., Kim, S., Distefano, A., Samuel, V. T., Neschen, S., Zhang, D., Wang, A., Zhang, X. M., Kahn, M., Cline, G. W., Pandey, S. K., Geisler, J. G., Bhanot, S., Monia, B. P., & Shulman, G. I. (2007). Suppression of diacylglycerol acyltransferase-2 (DGAT2), but not DGAT1, with antisense oligonucleotides reverses diet-induced hepatic steatosis and insulin resistance. The Journal of biological chemistry, 282(31), 22678–22688. https://doi.org/10.1074/jbc.M704213200

He, X., Sun, J., & Huang, X. (2018). Expression of caspase-3, Bax and Bcl-2 in hippocampus of rats with diabetes and subarachnoid hemorrhage. *Experimental and therapeutic medicine*, *15*(1), 873–877. <https://doi.org/10.3892/etm.2017.5438>

Jernberg, J.N., Bowman, C.E., Wolfgang, M.J. and Scafidi, S., 2017. Developmental regulation and localization of carnitine palmitoyltransferases (CPT s) in rat brain. *Journal of neurochemistry*, *142*(3), pp.407-419.

Long, C., Xiao, Y., Li, S., Tang, X., Yuan, Z., & Bai, Y. (2019). Identification of optimal endogenous reference RNAs for RT-qPCR normalization in hindgut of rat models with anorectal malformations. *PeerJ*, *7*, e6829. https://doi.org/10.7717/peerj.6829

Nakai, N., Obayashi, M., Nagasaki, M., Sato, Y., Fujitsuka, N., Yoshimura, A., Miyazaki, Y., Sugiyama, S., & Shimomura, Y. (2000). The abundance of mRNAs for pyruvate dehydrogenase kinase isoenzymes in brain regions of young and aged rats. *Life sciences*, *68*(5), 497–503. https://doi.org/10.1016/s0024-3205(00)00947-4

Wojcik, B., Miklosz, A., Zabielski, P., Chabowski, A., & Gorski, J. (2017). Effect of tachycardia on mRNA andf protein expression of the principal components of the lipolytic system in the rat's heart ventricles. *Journal of physiology and pharmacology : an official journal of the Polish Physiological Society*, *68*(5), 731–736.
